# Correlations of microRNA-124a and microRNA-30d with clinicopathological features of breast cancer patients with type 2 diabetes mellitus

**DOI:** 10.1186/s40064-016-3786-9

**Published:** 2016-12-22

**Authors:** Yu-Ling Han, Xian-E. Cao, Ju-Xun Wang, Chun-Ling Dong, Hong-Tao Chen

**Affiliations:** 1 Department of Breast and Thyroid Surgery, Linyi People’s Hospital, Linyi, 276000 People’s Republic of China; 2 Department of Geriatrics, Linyi People’s Hospital, North Park Road, 200 Meters East of Municipal Party School, Linyi, 276000 Shandong Province People’s Republic of China; 3 Linyi City Family Planning Management Station, Linyi, 276000 People’s Republic of China; 4 Department of Nurse, Linyi People’s Hospital, Linyi, 276000 People’s Republic of China; 5 Department of Rheumatism, Linyi People’s Hospital, Linyi, 276000 People’s Republic of China

**Keywords:** MicroRNA-124a, MicroRNA-30d, Breast cancer, Type 2 diabetes mellitus, Clinicopathological features

## Abstract

This study intends to investigate the correlations of miR-124a and miR-30d with clinicopathological features of breast cancer (BC) patients with type 2 diabetes mellitus (T2DM). A total of 72 BC patients with T2DM (diabetic group) and 144 BC patients without T2DM (non-diabetic group) were enrolled in this study. Blood glucose was detected by glucose oxidase methods. Glycosylated hemoglobin (HbA1c) was measured by high performance liquid chromatography. Fasting insulin (FIns) was measured by chemiluminescent microparticle immunoassay. Automatic biochemical analyzer was used to detect triglyceride, total cholesterol (TC), high-density lipoprotein cholesterol (HDL-C) and low-density lipoprotein cholesterol (LDL-C). Estradiol (E_2_) was detected by radioimmunoassay. Homeostasis model assessment was applied to assess the insulin resistance (HOMA-IR) and β-cell insulin secretion (HOMA-IS). The expressions of miR124a and miR-30d were measured by quantitative real-time polymerase chain reaction (qRT-PCR). There were significant differences in age, the ratio of menopause, body mass index (BMI), HDL-C, TC, 2-h plasma glucose (2hPG), FIns, HbA1c, HOMA-IS and HOMA-IR between the diabetic and non-diabetic groups. The diabetic group had higher incidence of lymph node metastasis than non-diabetic group. The miR-124a expression was down-regulated while the miR-30d expression was up-regulated in BC patients with T2DM. The correlation analysis showed that miR-124a expression was positively correlated with HDL-C, while it was negatively correlated with age, HbA1c, LDL-C and E_2_. However, the miR-30d expression was negatively correlated with HDL-C but positively correlated with age, HbA1c, LDL-C and E_2_. In conclusion, miR-124a and miR-30d may be correlated with clinicopathological features of BC patients with T2DM. The miR-124a and miR-30d could serve as novel biomarkers for early diagnosis of BC in patients with T2DM.

## Background

Breast cancer (BC) is one of the most common malignant neoplasms for females with 1.4 million new diagnoses a year worldwide, and is one of the leading causes of cancer-related death (Ban and Godellas 2014). Diabetes mellitus (DM), commonly referred to as diabetes, is a group of metabolic diseases with high blood sugar levels over a prolonged period, and more than 95% of all DM cases is type 2 diabetes mellitus (T2DM) (American Diabetes 2013, 2014). It is reported that approximately 7% of the people worldwide in the age between 20 and 79 years is estimated to have DM in 2010 and the number is expected to rise by more than 50% in the next 20 years (Ginter and Simko 2012). Further, studies focused on the DM have demonstrated that DM was implicated in various cancers development, including pancreas cancer, colon cancer, liver cancer, esophagus cancer, endometrial cancer and BC (Chang et al. 2012; Pan et al. 2012; Shikata et al. 2013; Wang et al. 2012). The relative risk for mortality of BC patients has been reported to be twofold greater in BC patients with DM than in those without DM (Liao et al. 2011), and the epidemiologic evidences showed that DM patients have a significant higher risk of BC and is closely associated with a poor prognosis of BC patients (Luo et al. 2014; Oppong et al. 2014). The BC patients with T2DM had been reported to be more likely to associate with a higher incidence of lymph nodes and were at an advanced tumor stage, indicating a shorter survival time (He et al. 2015).

MicroRNAs (miRs) have been reported to be implicated in various malignancies and involved in a variety of biological processes, including cell proliferation, differentiation, apoptosis, and metastasis (Di Leva et al. 2014; Farazi et al. 2013). Acting as an abundant miR in the central neuron system, miR-124a has been reported to be linked to the progression of various tumors and it may act as an important regulator of the transcriptional protein network in beta-cells responsible for regulating intracellular signaling (Baroukh et al. 2007; Chen et al. 2013). Overexpression of miR-124a may inhibit the glucose-stimulated insulin secretion and the altered expression of miR-124a may lead to beta-cell dysfunction in T2DM patients (Sebastiani et al. 2015). It has been reported that the methylation of miR-124a in adjacent normal mucosa may be correlated with the microsatellite instability of colorectal cancer (Deng et al. 2011). Further, miR-30d regulates various physiological processes in normal tissues or cancer cells, including development, metastasis, apoptosis, senescence, proliferation and differentiation (Bridge et al. 2012; Zhao et al. 2012). MiR-30d may be acted as a novel oncogene that may be implicated in the development of tumors and homeostasis, and may be served as a potential useful biomarker or drug target in human malignancies (Yang et al. 2013). MiR-30d also plays a key role in activating glucose-induced insulin gene transcription and in avoiding beta-cell functions impaired by pro-inflammatory cytokines, which may act as a potential target for diabetes intervention (Zhao et al. 2012).

Indeed, both miR-124a and miR-30d may participate in the development of BC and T2DM occurrence. In the present study, we investigated the expressions of miR-124a and miR-30d in BC patients with T2DM, and analyzed correlations of miR-124a and miR-30d with clinicopathological features of BC patients with T2DM.

## Methods

### Study subjects

Between January 2012 and January 2015, a total of 72 patients diagnosed as BC with T2DM at Linyi People’s Hospital were enrolled as diabetic group in this study. According to the principle of 1:2, 144 patients diagnosed as BC without T2DM were randomly recruited as non-diabetic group using the random number table. The matching principle was the date of hospital visiting ±1 month. All patients were confirmed as BC patients through the paraffin slide biopsy via the Department of pathology. The diagnosis of T2DM was as following: (1) patients were inquired of medical history (diagnosed as T2DM by secondary-and tertiary-level hospitals) by physician preoperative, medication history and the condition of blood sugar monitoring; (2) fasting blood glucose (FPG) ≥7.0 mmol/L at admission or random blood glucose levels ≥11.11 mmol/L, or the 2-h plasma glucose (2hPG) from an oral glucose tolerance test (OGTT) ≥11.1 mmol/L. Inclusion criteria: all patients had a complete clinical data, including age, body mass index (BMI), family history, past medical history, menopausal status, tumor size, axillary lymph nodes, etc. Exclusion criteria: (1) BC patients with type 1 diabetes mellitus or secondary diabetes mellitus; (2) male BC patients; (3) patients with bilateral BC; (4) patients with carcinoma in situ of breast or stage IV BC patients; (5) BC patients who had received neoadjuvant chemotherapy or estrogen therapy; (6) patients with incomplete clinical data; and (7) patients with nonstandard anticancer therapy. Among the 72 BC patients with T2DM, most of them were treated with oral hypoglycemic agents, but the patients with poor control of glucose level before operation were treated with insulin therapy after physician consultation from Department of endocrinology. All the patients enrolled in the study were underwent unilateral modified radical mastectomy, and received 6 cycles of TAC chemotherapy (Taxotere + Adriamycin + cyclophosphamide) postoperatively. The BC patients with lymph nodes were received postoperative radiotherapy, and the patients with hormone-receptor-positive BC who received endocrine therapy. All patients were appropriately informed about this study and signed their informed consents forms. This study was conducted with the approval of the ethics committee of Linyi People’s Hospital, and the ethical approval for this study conformed to the standards of the Declaration of Helsinki (Pn 2014).

The medical records were reviewed, including the admission number, name, age, BMI, menopausal status, history of diabetes, family history of BC, past medical history, diameter of tumor, pathological types, metastasis of axillary lymph nodes, tumor stage, histological grade, immunohistochemical markers (estrogen receptor: ER; progesterone receptor: PR; human epithelial growth factor receptor 2: Her-2, P-glyprotein: P-gp; topoisomerase II: Topo-II; glatocnine-S-tranferase-π: GST-π), surgical procedures, operation time, chemotherapy regimens and course of chemotherapy. All patients were followed up after surgery. The tumor node metastasis (TNM) stages were classified in accordance with the grading standard published by the American Joint Committee on Cancer (AJCC) or Union for International Cancer Control (UICC) (Singletary et al. 2003).

### Biochemical parameters

Blood glucose was determined by glucose oxidase method using a Roche glucometer (Accu-Chek Active, Roche Ltd., Germany). Glycosylated hemoglobin (HbA1c) was measured by high performance liquid chromatography (ADAMSTMA1c HA-8160, Japan). Fasting insulin (FIns) was detected by chemiluminescent microparticle immunoassay (CMIA; Abbott i2000SR, USA). Triglyceride (TG), total cholesterol (TC), high-density lipoprotein cholesterol (HDL-C) and low-density lipoprotein cholesterol (LDL-C) were detected by automatic biochemical analyzer (Roche Ltd., Germany). Homeostasis model assessment was applied to assess insulin resistance and β-cell insulin secretion, i.e. HOMA-IR = (FPG × FIns)/22.5. HOMA of β-cell insulin secretion (HOMA-IS) = 20 × FIns/(FPG − 3.5).

The last menstrual periods of patients were recorded, and the concentration of E_2_ in the follicular phase at 2–4 days after menstruation was detected. The E_2_ level in the blood serum was measured by radioimmunoassay (radio-immunity kits, Depp Biological Technology and Medical Products Co. Ltd, Tianjin, China), with intra-assay coefficient of variance (CV) 7.96% and inter-assay CV 9.22%.

### Quantitative real-time polymerase chain reaction (qRT-PCR)

Blood sample from the each patient after 12 h fasting was collected in the next morning, and was centrifuged following the instructions of RNA extraction kit. Total RNA was extracted by a miRNeasy Mini Kit (Qiagen Company, Hilden, Germany). RNA samples (5 μL) were diluted 20 times in RNA-free ultrapure water. The concentration and quality of RNA were determined by the ultraviolet absorbance at 260 and 280 nm (optical density, OD; OD260/OD280 ratio) using an ultraviolet spectrophotometer. The OD260/OD280 ratio between 1.7 and 2.1 indicated that the RNA had high purity, which could meet the requirements of further research processes. The cDNA template was generated by reverse transcription in a PCR amplifier. The qRT-PCR was conducted by ABI 7500 quantitative PCR System (Life Technologies, USA). The reaction condition was 40 cycles of denaturation at 95 °C for 10 min, denaturation at 95 °C for 10 s, annealing at 60 °C for 20 s and extension at 72 °C for 34 s. The primers were synthesized by Sangon Biotech (Shanghai, China), as illustrated in Table 1. U6 snRNA was used as an internal control. The cycle number at threshold (Ct value) was used to calculate the relative expressions of miR124a and miR30d. The results were presented as fold change, and calculated using the 2^−ΔΔCT^ method (Livak and Schmittgen 2001), with the formula as: ΔΔCT = ΔCt_diabetic group_ –ΔCt_non-diabetic group_, ΔCt = Ct_miR_ – Ct_U6_. The experiments were totally repeated for 3 times.

**Table 1:** The primer sequences for qRT-PCR

Gene	Forward (5′-3′)	Reverse (5′-3′)
U6	GCTTCGGCAGCACATATACTAAAAT	CGCTTCACGAATTTGCGTGTCAT
miR-124a	UUAAGGCACGCGGUGAAUGCCA	CTTAAGGCACGCGGTGAATGCCA
miR-30d	UGUAAACAUCCCCGACUGGAAG	TGTAAACATCCCCGACTGGAAGA

*qRT-PCR* quantitative real-time polymerase chain reaction

### Statistical analysis

SPSS19.0 was used to conduct the statistical analysis. Measurement data were expressed as mean ± standard deviation (SD). Variance homogeneity test was used before analysis, and One-Way ANOVA analysis was used for multiple group comparisons. The least significant difference (LSD)-*t* test or *Chi square test* was used in pairwise comparison of averages among groups. The relationships between the expressions of miR-124a and miR-30d and clinicopathological features and biochemical parameters were analyzed by Pearson correlation analysis and linear regression analysis. *P* < 0.05 showed statistically significant.

## Results

### Comparisons of clinical features and biochemical parameters between the diabetic group and non-diabetic group

The mean age of patients in the diabetic group was 52.72 ± 6.23 years, and the mean BMI was 24.68 ± 4.74 kg/m^2^. In the diabetic group, 33.61% cases were premenopausal patients and 76.39% cases were postmenopausal patients. The mean age of patients in the non-diabetic group was 50.08 ± 4.76 years and the mean BMI was 23.21 ± 3.25 kg/m^2^. Of the 144 patients in the non-diabetic group, 44.44% cases were premenopausal patients and 55.56% cases were postmenopausal patients. There were significant differences on age, the ratio of menopause and BMI between the two groups (age: *P* < 0.001; ratio of menopause; *P* = 0.003; BMI: *P* = 0.008). Further, significant differences were observed in HDL-C, LDL-C, TC, FPG, 2hPG, FIns, HbA1c, HOMA-IS, HOMA-IR and E_2_ between diabetic group and non-diabetic group (all *P* < 0.05). The family history of BC, underlying diseases, systolic blood pressure (SBP), diastolic blood pressure (DBP) or TG had no significant differences between the two groups (all *P* > 0.05), as illustrated in Table 2.

**Table 2:** Comparisons of clinical features and biochemical parameters between diabetic group and non-diabetic group

	Diabetic group (n = 72)	Non-diabetic group (n = 144)	*t*/χ^2^	*P* value
Age (years)	52.72 ± 6.23	50.08 ± 4.76	3.113	<0.001
Family history of BC	5.56% (4)	4.17% (6)	0.458	0.647
Underlying diseases	19.44% (14)	20.83% (30)	0.057	0.811
Ratio of menopause	76.39% (55)	55.56% (80)	8.889	0.003
BMI (Kg/m^2^)	24.68 ± 4.74	23.21 ± 3.25	2.673	0.008
SBP (mmHg)	124.68 ± 10.35	125.36 ± 11.23	0.430	0.667
DBP (mmHg)	78.36 ± 7.93	80.57 ± 8.54	1.835	0.068
HDL-C (mmol/L)	1.21 ± 0.32	1.31 ± 0.36	1.995	0.047
LDL-C (mmol/L)	3.85 ± 0.74	3.12 ± 0.65	7.425	<0.001
TG (mmol/L)	1.32 ± 0.35	1.41 ± 0.41	1.594	0.112
TC (mmol/L)	4.45 ± 0.87	3.64 ± 0.68	7.498	<0.0001
FPG (mmol/L)	8.32 ± 0.73	5.27 ± 0.43	38.56	<0.001
2hPG (mmol/L)	14.68 ± 1.02	5.58 ± 0.47	89.810	<0.001
HbA1c (%)	10.57 ± 2.11	5.64 ± 1.57	19.320	<0.001
FIns (uU/mL)	10.14 ± 3.25	5.33 ± 1.87	6.954	<0.001
HOMA-IS	42.84 ± 14.53	60.50 ± 16.29	7.779	<0.001
HOMA-IR	3.75 ± 1.25	1.27 ± 0.50	20.750	<0.001
E_2_ (pg/mL)	50.20 ± 11.40	23.66 ± 8.63	19.080	<0.001

Comparisons on family history of BC, underlying diseases and the ratio of menopause between the two groups were measured by χ^2^ test; BMI, weight (kg)/height (m^2^); Normal BMI, 18.5–22.9 kg/m^2^

Diabetic group, breast cancer patients with type 2 diabetes mellitus; Non-diabetic group, breast cancer patients without type 2 diabetes mellitus

*BC* breast cancer, *BMI* body mass index, *SBP* systolic blood pressure, *DBP* diastolic blood pressure, *HDL*-*C* high-density lipoprotein cholesterol, *LDL*-*C* low-density lipoprotein cholesterol, *TG* triglyceride, *TC* total cholesterol, *FPG* fasting blood glucose, *2hPG* 2-hour postprandial blood glucose, *HbA1c* glycosylated hemoglobin, *FIns* fasting insulin, *HOMA*-*IR* homeostasis model assessment of insulin resistance, *HOMA*-*IS* HOMA of β-cell insulin secretion, *E*_*2*_ estradiol

### Comparisons of pathological features between the diabetic group and non-diabetic group

The comparisons of pathological features indicated that diabetic group had higher incidence of lymph node metastasis than non-diabetic group (*P* < 0.001). However, there were no significant differences in diameter of tumor, ratio of axillary lymph nodes, pathological types, tumor stage, histological grade, ER, PR, Her-2, P-gp, Topo-II and Gst-π (all *P* > 0.05) (Table 3).

**Table 3:** Comparisons of pathological characteristics between diabetic group and non-diabetic group

Characteristic	Diabetic group (n = 72) [%]	Non-diabetic group (n = 144) [%]	χ^2^	*P* value
T stage				
T1	16 (22.2)	36 (25.0)	0.809	0.667
T2	35 (48.6)	74 (51.4)		
T3	21 (29.2)	34 (23.6)		
Axillary lymph nodes				
Positive	45 (62.5)	75 (52.1)	2.109	0.146
Negative	27 (37.5)	69 (47.9)		
Pathological types				
Invasive ductal carcinoma	66 (91.7)	136 (94.4)	0.890	0.828
Invasive lobular carcinoma	3 (4.2)	3 (2.1)		
Invasive papilloma	2 (2.8)	3 (2.1)		
Mucinous adenocarcinoma	1 (1.4)	2 (1.4)		
Tumor stage				
I	4 (5.6)	19 (13.2)	2.966	0.227
II	27 (37.5)	51 (35.4)		
III	41 (56.9)	74 (51.4)		
Histological grade				
WHO I	5 (6.9)	9 (6.2)	2.099	0.350
WHO II	42 (58.3)	98 (68.1)		
WHO III	25 (34.7)	37 (25.7)		
Lymph node metastasis				
Positive	45 (62.5)	55 (38.2)	11.410	<0.001
Negative	27 (37.5)	89 (61.8)		
ER				
Positive	42 (58.3)	88 (61.1)	0.155	0.694
Negative	30 (41.7)	56 (38.9)		
PR				
Positive	40 (55.6)	91 (63.2)	1.174	0.279
Negative	32 (44.4)	53 (36.8)		
Her-2				
Positive	25 (34.7)	66 (458)	2.431	0.119
Negative	47 (65.3)	78 (54.2)		
P-gp				
Positive	60 (83.3)	121 (84.0)	0.017	0.896
Negative	12 (16.7)	23 (16.0)		
Topo-II				
Positive	64 (88.9)	132 (91.7)	0.441	0.507
Negative	8 (11.1)	12 (8.3)		
Gst-π				
Positive	39 (54.2)	70 (48.6)	0.593	0.441
Negative	33 (45.8)	74 (51.4)		

Diabetic group, breast cancer patients with type 2 diabetes mellitus; non-Diabetic group, breast cancer patients without type 2 diabetes mellitus

*WHO* World Health Organization, *ER* estrogen receptor, *PR* progesterone receptor, *Her*-*2* human epithelial growth factor receptor 2, *P*-*gp* P-glyprotein, *Topo*-*II* topoisomerase II, *Gst*-*π* glatocnine-S-tranferase-π

### Expressions of miR-124a and miR-30d in the diabetic group and non-diabetic group

**Fig. 1:**
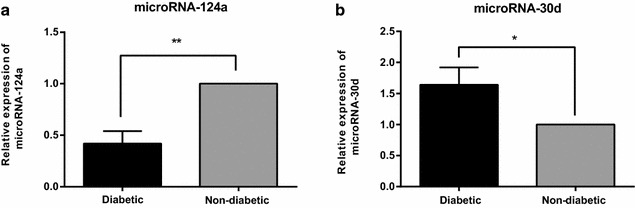
Expressions of miR-124a and miR-30d in the diabetic group and non-diabetic group. *Note* **a** miR-124a expression in the diabetic and non-diabetic groups; **b** miR-30d expression in the diabetic and non-diabetic groups; *P < 0.05; **P < 0.05; miR-124, microRNA-124a; miR-30d, microRNA-30d

The expressions of miR-124a and miR-30d in the diabetic group and non-diabetic group were detected by qRT-PCR. The results showed that the miR-124a expression in the diabetic group was 0.42 fold of that in the non-diabetic group, as the relative expression of miR-124a in the non-diabetic group was defined as 1 (*P* < 0.05). However, the miR-30d expression in the diabetic group was 1.64 fold of that in the non-diabetic group, as the relative expression of miR-30d in the non-diabetic group was defined as 1 (*P* < 0.05) (Fig. 1). These results suggested that miR-124a may act as an anti-oncogene while the miR-30d may act as an oncogene in the development of BC with T2DM.

### Correlations of miR-124a and miR-30d with clinicopathological features in BC patients with T2DM

The Pearson correlation analysis was conducted to explore the correlations between miRs124a/30d and age, BMI, FPG, HbA1c, 2hPG, TC, HDL-C and LDL-C. The correlation analysis showed that the miR-124a expression was positively associated with HDL-C (*P* < 0.001), while it was negatively associated with age, HbA1c, LDL-C and E_2_ (all *P* < 0.05). No significant differences were observed between the miR-124a expression and BMI, FPG, 2hPG or TC (all *P* > 0.05). Further, the miR-30d expression was negatively correlated with HDL-C (*P* < 0.001), while it was positively correlated with age, HbA1c, LDL-C and E_2_ (all *P* < 0.05). There were no significant differences between the miR-30d expression and BMI, FPG, 2hPG or TC (all *P* > 0.05), as shown in Table 4. Linear regression analysis showed that the HbA1c, LDL-C, HDL-C and E_2_ were independent factors for expressions of miR-124a and miR-30d (Tables 5, 6).

**Table 4:** Correlation analysis of miR-124a and miR-30d with various biochemical parameters in BC patients with T2DM

Index	miR-124a		miR-30d	
	r value	*P* value	r value	*P* value
Age (years)	−0.353	0.002	0.333	0.004
Ratio of menopause	−0.049	0.685	0.134	0.262
BMI (Kg/m^2^)	−0.172	0.148	0.218	0.066
HDL-C (mmol/L)	0.698	<0.001	−0.731	<0.001
LDL-C (mmol/L)	−0.754	<0.001	0.786	<0.001
TC (mmol/L)	−0.071	0.554	0.069	0.564
FPG (mmol/L)	−0.115	0.337	0.097	0.419
2hPG (mmol/L)	−0.097	0.418	0.091	0.447
HbA1c (%)	−0.443	<0.001	0.496	<0.001
FIns (uU/mL)	−0.139	0.246	0.128	0.284
HOMA-IS	−0.085	0.478	0.096	0.421
HOMA-IR	−0.160	0.179	0.139	0.243
E_2_ (pg/mL)	−0.763	<0.001	0.765	<0.001

*BMI* body mass index, *HDL*-*C* high-density lipoprotein cholesterol, *LDL*-*C* low-density lipoprotein cholesterol, *TC* total cholesterol, *FPG* fasting blood glucose, *2hPG* 2-hour postprandial blood glucose, *HbA1c* glycosylated hemoglobin, *FIns* fasting insulin, *HOMA*-*IR* homeostasis model assessment of insulin resistance, *HOMA*-*IS* HOMA of β-cell insulin secretion, *E*_*2*_ estradiol

**Table 5:** Linear regression analysis of the factors for miR-124a expression

Model	Unstandardized coefficients		Standardized coefficients	t	Sig.
	B	Std. error	Beta		
(Constant)	0.866	0.108		8.025	<0.001
LDL-C	−0.045	0.015	−0.275	−2.888	0.005
Age	−0.002	0.001	−0.125	−1.977	0.052
HDL-C	0.098	0.030	0.263	3.306	0.002
HbA1c	−0.008	0.004	−0.145	−2.222	0.030
E_2_	−0.004	0.001	−0.341	−3.744	<0.001

HDL-C, high-density lipoprotein cholesterol; LDL-C, low-density lipoprotein cholesterol; HbA1c, glycosylated hemoglobin; E_2_, estradiol; B: regression coefficient; *Std. error*: standard error of regression; Sig: significance, *P* value

**Table 6:** Linear regression analysis of the factors for miR-30d expression

Model	Unstandardized coefficients		Standardized coefficients	t	Sig.
	B	Std. error	Beta		
(Constant)	0.617	0.223		2.764	0.007
LDL-C	0.121	0.032	0.321	3.806	<0.001
Age	0.004	0.003	0.098	1.755	0.084
HDL-C	−0.252	0.062	−0.288	−4.089	<0.001
HbA1c	0.026	0.008	0.194	3.364	0.001
E_2_	0.007	0.002	0.287	3.561	0.001

*HDL*-*C* high-density lipoprotein cholesterol, *LDL*-*C* low-density lipoprotein cholesterol, *HbA1c* glycosylated hemoglobin, *E*_*2*_ estradiol, *B* regression coefficient, *Std. error* standard error of regression, *Sig* significance, *P* value

## Discussion

In the present study, we aimed to explore the correlations of miR-124a and miR-30d with BC patients with T2DM. We explore the correlations of miR-124a and miR-30d with the clinicopathological features of BC patients with T2DM. We found that the miR-124a expression was positively associated with HDL-C, while miR-30d expression was negatively correlated with HDL-C. Moreover, miR-124a expression was negatively associated with age, HbA1c, LDL-C and E_2_, while miR-30d expression was positively correlated with age, HbA1c, LDL-C and E_2_. These results indicated that the levels of HbA1c, LDL-C, HDL-C and E_2_ were correlated with the expressions of miR-124a and miR-30d, and may be acted as independent factors for expressions of miR-124a and miR-30d.

It was well-known that T2DM is characterized by insulin resistance and impaired insulin secretion caused by insufficiency of pancreatic beta-cells (Mizokami-Stout et al. 2012; Okuno et al. 2013). Additionally, HbA1c may capture the glucose exposure that may relevant to cancer risk and higher HbA1c level may be correlated with a higher risk of cancer incidence and cancer-related mortality (Joshu et al. 2012; Li et al. 2014). Estrogen has also been reported to be involved in the pathogenesis and disease progression of BC and down-regulated estrogen level could be a potential management for most patients with estrogen responsive tumors (Su et al. 2013). Further, the estrogen may promote cell proliferation and inhibits cell apoptosis by modulating gene transcription in estrogen-dependent tumors, and the high serum E_2_ levels may be associated with specific gene expression patterns in BC tissues (Chalasani et al. 2014; Kim et al. 2013).

Our study results have revealed that the miR-124a expression in BC patients with T2DM was significantly lower than that in BC patients without T2DM. The tumor-related miRs function as tumor suppressors or oncogenes and regulate various aspects of carcinogenesis, including cell proliferation, cell-cycle control, metastasis, and angiogenesis (Landskroner-Eiger et al. 2013; Profumo and Gandellini 2013). MiR-124a is mainly expressed in brains and pancreas, and the over expression of miR-124a in pancreatic β-cells can improve the insulin secretion, but it can reduce the insulin secretion stimulated by high concentration of glucose (Baroukh et al. 2007). The expression and mechanisms of miR-124 have also been investigated in BC, and Han et al. (2013) have found that miR-124 may play a key role in inhibiting the invasion and metastasis of BC cells, probably by directly targeting *CD151* genes. MiR-124a overexpression could down-regulate FoxA2 expression, which could bind with PDX1 and ISL1 in the islet amyloid polypeptide (IAPP) promoter, thus decreasing the IAPP levels and inhibiting the apoptosis of β-cells (Jing et al. 2014). Further, Li et al. (2013) have demonstrated that the expression of miR-124 was down-regulated in BC patients, and the miR-124 might be acted as a tumor suppressor in BC through the regulation of *FLOT1* gene. The study performed by Dong et al. (2015) has revealed that decreased expression of miR-124 may be associated with advanced TNM stage, lymph node metastasis and poorer pathological differentiation, implying that down-regulation of miR-124 may be an independent unfavorable prognostic factor for BC patients. Further, high levels of insulin are mitogenic for BC cells, and overexpressed insulin receptors are often found in BC patients (Kaplan et al. 2012). Hyperexpression of miR-124a may be impaired glucose-stimulated insulin secretion, and the silencing of the miR-124a resulted in increased expression of target genes for beta-cell function, indicating that an altered miR-124a expression may lead to beta-cell dysfunction in T2DM (Sebastiani et al. 2015). Meanwhile, insulin release from pancreatic beta-cells acts an important role in blood glucose homeostasis, and the pancreatic development is a complex sequential expression of a gamut of transcription factors. Foxa2 deficiency may result in excessive insulin release in response to amino acids and complete loss of glucose-stimulated insulin secretion (Gao et al. 2010; Lantz et al. 2004). Baroukh et al. (2007) have demonstrated that miR-124a may be implicated in the cell differentiation process of beta-cells, and the miR-124a may play as a regulator of a key transcriptional protein network in beta-cells responsible for modulating intracellular signaling by targeting *Foxa2* gene. In this regard, we suspected that the lower expression of miR-124a may be involved in the development and progression of BC patients combined with T2DM.

In this study, we found that the miR-30d expression in BC patients with T2DM was significantly higher than that in BC patients without T2DM. Meanwhile, we found that miR-30d expression was positively associated with the levels of HbA1c, LDL-C and E_2_, and the levels of HbA1c, LDL-C and E_2_ may be acted as independent factors for expression of miR-30d. Recent evidence has suggested that miR-30d may be acted as a novel antioncogene (Li et al. 2012; Lu et al. 2009; Zhao et al. 2012). In patients with diabetes or with high level of glucose, miR-30d could regulate *Map4k4* expression to increase the levels of insulin transcription factors, thus promoting the insulin secretion and reducing TNF-α-induced transcription and production of insulin genes (Tang et al. 2009). It has been reported that miR-30d may suppress renal carcinoma cell proliferation by regulating cyclin E2 expression at a post-transcriptional level (Yu et al. 2014). Tang et al. (2009) have found that the up-regulated expression of miR-30d by glucose may increase the insulin gene expression, while the inhibition of miR-30d may abolish glucose-stimulated insulin gene transcription, which may be a negative regulator of insulin gene expression.

## Conclusion

MiR-124a and miR-30d may be correlated with clinicopathological features of BC patients with T2DM. The miR-124a and miR-30d could serve as novel biomarkers for early diagnosis of BC in patients with T2DM. However, the exact mechanism of miR-124a and miR-30d in the progression of BC combined with T2DM was still unclear. Further study based on the target genes of miR-124a or miR-30d need to be conducted to explore the underlying mechanisms of miR-124a and miR-30d on the development of BC combined with T2DM.
